# The Influence of Sample Microfabrication and Annealing on the Mechanical Strain–Stress Behavior of Stainless Steels and Corrosion Resistant Aluminum Alloys in Micro-Tensile Tests

**DOI:** 10.3390/mi16030309

**Published:** 2025-03-06

**Authors:** Janko Auerswald, Joel Tenisch, Christoph Fallegger, Markus Seifert

**Affiliations:** 1 Lucerne School of Engineering and Architecture, Lucerne University of Applied Sciences and Arts, 6048 Horw, Switzerland; 2 Research and Development Department, ZwickRoell, 89079 Ulm, Germany

**Keywords:** micro-tensile test, strain–stress behavior, shear band zone, mechanical properties, austenitic stainless steel, aluminum alloy, microwaterjet, laser cutting, annealing

## Abstract

Miniaturized components for enhanced integrated functionality or thin sheets for lightweight applications often consist of face-centered cubic metals. They exhibit good strength, corrosion resistance, formability and recyclability. Microfabrication technologies, however, may introduce cold work or detrimental heat-induced lattice defects into the material, with consequences for the mechanical properties. Austenitic stainless steels (1.4310, 1.4301) and aluminum alloys (EN AW-5005-H24, EN AW-6082-T6) were selected for this study. The influence of pulsed fiber laser cutting, microwaterjet cutting, and annealing on the strain–stress behavior was investigated. The micro-tensile test setup comprised a flex-structure force sensor, a laser extensometer, and a dedicated sample holder. Fiber laser cut 1.4310 samples exhibited early failure at low fracture strain in narrow shear band zones. The shear band zones were detectable on the sample surface, in the laser extensometer images, in the horizontal sections of the stress–strain curves, and in the microstructure. Inside the shear band zones, grains were strongly elongated and exhibited numerous parallel planar defects. Heat-induced chromium carbides, in combination with low stacking fault energy (SFE) and elevated carbon content, favored shear band zone formation in 1.4310. In contrast, microwaterjet cut high SFE materials EN AW-5005-H24 and EN AW-6082-T6, as well as low-carbon austenitic stainless steel 1.4301, exhibited uniform plastic deformation.

## 1. Introduction

The trends towards more sustainability, component durability, reduction in weight, and enhanced integrated functionality have triggered a significant innovation boost in microtechnology and materials science. Interesting target applications range from (electro-)mobility, transportation, aerospace, shipping, and food packaging industries to micromechanical systems, portable consumer electronics, point-of-care devices, and watches. The mechanical strain–stress behavior of miniaturized components made of face-centered cubic (fcc) alloys represents an important research topic in this context. Typical fcc materials used for these applications include austenitic stainless steel and aluminum alloys. They provide good corrosion resistance, strength, formability, and recyclability.

**Figure 1:**
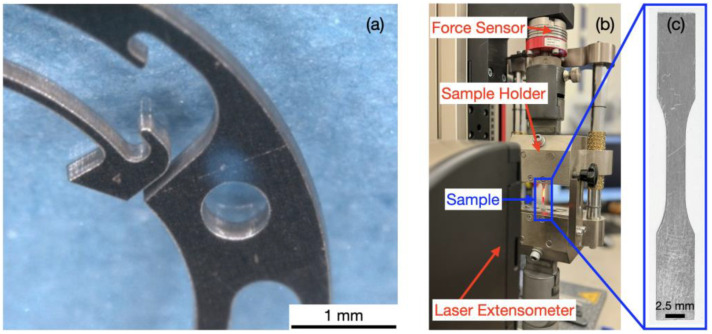
(**a**) An example of a micromechanical spring component made of 1.4310. (**b**) The micro-tensile test setup. (**c**) A typical micro-tensile test sample (here, stainless steel 1.4310, 100 μm thick).

The emphasis of this investigation is placed on the alloy 1.4310, an austenitic stainless steel. It is used for miniaturized spring components in micromechanical systems, e.g., in watch movements for day, date, and month switching mechanisms (Figure 1a), or for precise positioning mechanisms based on flex structures. Its elevated carbon content provides a high material strength, which can be further increased by strain-induced martensite [1]. The increase in strength of 1.4310 comes at a still acceptable corrosion resistance and cost compared to low carbon 1.4301, which is the standard austenitic stainless sheet steel. It exhibits better corrosion resistance and ductility than 1.4310, but also lower material strength. Cross-section reduction in the corrosion-resistant 1.4301 sheets has also become a major issue in the recent years, e.g., in the appliance industry, where sheet thickness has decreased to 0.6 mm and less. Aluminum alloys of the series 5xxx (Al-Mg, not oversaturated, i.e., <3 wt% Mg) and 6xxx (Al-Si-Mg) also provide good corrosion resistance, strength and formability; however, at a lower temperature resistance and stiffness than austenitic stainless steels. Their advantage consists of a reduced specific weight. Alloy EN AW-5005-H24 is work-hardened and partially annealed. It represents one of the standard sheet materials in lightweight construction, e.g., for buses, trucks, and trailers, for boat and ship components, or for non-magnetic electrical enclosures. Alloy EN AW-6082-T6 exhibits an increased strength due to precipitation hardening. It is used in (electro-)mobility for automotive chassis components or in the aerospace industry for interior support structures, e.g., in medical rescue helicopters and airplanes. The four selected materials in this investigation are representative of the numerous similar fcc stainless steels and aluminum alloys.

Plastic deformation in fcc metals at room temperature is based either on the dislocation glide or on the formation of partial dislocations and the corresponding planar defects. A full dislocation glide in fcc metals occurs on the {111} slip planes, with atoms moving in ½⋅⟨110⟩ directions at the dislocation line. The split of a full dislocation into two partial dislocations leads to stacking faults between the two partial dislocations. The normal stacking sequence (ABCABC) of the closest-packed {111} slip planes is disturbed in this area. The formation of stacking faults occurs either if not enough favorably oriented slip systems are active, or if the stacking fault energy (SFE) of the material is low [2,3,4]. Depending on their number and sequence, several overlapping stacking faults in low SFE materials may result in parallel stacking fault bundles, twinning, or even shear bands.

Shear band zones are locally confined regions of massive plastic deformation and material weakening. On a mesoscopic scale, they occur under non-crystallographic angles and involve a multitude of grains [5]. On a microstructure scale, shear bands in γ-austenite of fcc stainless steels with low SFE are formed by multiple overlapping stacking faults. The stacking sequence irregularities of the {111} slip planes may result in hexagonal close-packed (hcp) ε-martensite, twinning, or random stacking fault bundles, depending on the actual faulted stacking sequence. Furthermore, crossing the shear bands may lead to α′-martensite formation in the shear band intersection regions of the microstructure [6]. Both ε-martensite and α′-martensite are less ductile, i.e., more brittle, than the fcc γ-austenite matrix. That makes shear band zones the preferred region of material failure. This is particularly problematic for miniaturized components with thin cross-sections.

Stacking fault energy has a significant influence on the deformation behavior of fcc metals and alloys. In fcc metals and alloys with high SFE, dislocation glide is by far the dominant slip mechanism. Full dislocations can easily change slip planes (cross-slip). This facilitates plastic deformation even at high plastic strains or at high concentrations of lattice defects in the microstructure [7]. In contrast, the fcc metals and alloys with low SFE are susceptible to the formation of partial dislocations and stacking faults. This results in irregularities of the {111} plane stacking sequence or twinning. High SFE materials comprise aluminum and its alloys (with typical SFE values of 160–250 mJ/m^2^). Low and medium SFE materials comprise austenitic stainless steels (with typical SFE values smaller than 25 mJ/m^2^), but also copper and its alloys [4,8,9,10,11]. The SFE of the fcc alloys also depends on the type and concentration of the alloying elements in the base metal. In fcc stainless steels, low SFE (<20 mJ/m^2^) favors the phase transformation of austenite to ε- and α′-martensite (transformation-induced plasticity, TRIP). Medium SFE (20–45 mJ/m^2^) leads to twinning (twinning-induced plasticity, TWIP). Higher SFE (>45 mJ/m^2^) favors plastic deformation by full dislocation glide [12,13].

However, the strain–stress behavior of the miniaturized components does not only depend on the material itself, but also on microfabrication technologies. Cold rolling or abrasive microwaterjet cutting may introduce cold work lattice defects. They reduce material stiffness, ductility, and corrosion resistance. They can be partially removed by annealing. Laser cutting generates heat-affected zones (HAZ). This may result in temper colors, metallurgical processes (e.g., chromium carbide formation in stainless steels or overaging of precipitation-hardened alloys), material embrittlement, or various corrosion issues. Thermally induced material modifications cannot be eliminated at typical moderate annealing temperatures.

The objective of this investigation was to study the influence of the selected microfabrication methods and annealing heat treatments on the deformation behavior and mechanical properties of the miniaturized 1.4310, 1.4301, EN AW-5005-H24, and EN AW-6082-T6 samples. Stress–strain curves were measured using dedicated, recently developed micro-tensile test equipment (Figure 1b) and procedures. Precise determination of small amounts of strain and stress, as well as accurate clamping and mechanical guiding, were key factors in this context. Microstructure analysis was employed to discuss the pronounced shear band zone formation in laser cut 1.4310 stainless steel samples.

## 2. Materials and Methods

Stainless steel bands (1.4310, 1.4301) were provided by Lamineries Matthey, Switzerland. Thin aluminum alloy sheets (EN AW-5005-H24, EN AW-6082-T6) were provided by Kiener + Wittlin, Switzerland and by an automotive industry manufacturer ZF, Germany. Table 1 and Table 2 show the chemical compositions of 1.4310 [14], 1.4301 [15], EN AW-5005 [16], and EN AW-6082 [17] in weight-percent, respectively.

**Figure 2:**
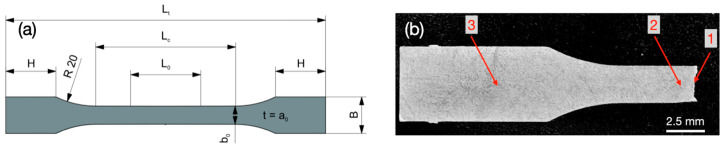
(**a**) The schematic design of the micro-tensile test sample geometry. (**b**) Mounted, polished, and etched 1.4310 sample after failure in micro-tensile test with 1—fracture zone, 2—deformation zone (large amount of plastic deformation) and 3—clamping area (larger width, little plastic deformation).

The design rules proposed in ISO 6892-1 [18] for standard size samples were considered for the geometries of the miniaturized micro-tensile test samples (Figure 2a and Table 3). The design rules and test parameters of the ASTM E8/E8M [19] standard are slightly different and were not considered in this study. However, the determination of material characteristics is similar in both standards. The micro-tensile test samples needed to exhibit a practicable stiffness and strength. Stainless steel samples (1.4310, 1.4301) were 100 μm thin. The thickness of the aluminum alloy samples amounted to 500 μm for EN AW-5005-H24 (simulating thin sheet-like micro components) and to 1 mm for EN AW-6082-T6 (simulating stronger beam-like micro components).

Micro-tensile test samples were fabricated using two different cutting techniques. “Cold” abrasive water jet micromachining was performed using a dedicated manufacturing system (Microwaterjet, Waterjet AG, Aarwangen, Switzerland) at a water pressure of 3200–3600 bar, with 220 mesh or finer garnet sand, and a maximum jet diameter of 500 μm. For laser cutting, a pulsed fiber laser system (YDFLP-8.50-LP-L-R, Shenzen JPT Opto-Electronics Co., Ltd., Shenzen, China) was employed. The beam source was a fiber laser with a pulse duration of 200 ns, a maximum average output power of 50 W, repetition rates from 1 to 600 kHz, a maximum pulse energy of 1.25 mJ at low repetition rates, a beam quality of M^2^ < 1.8 and a wavelength of 1064 nm. The scanner optics comprised a 200 mm focal length F-Theta lens, with a maximum scan speed of 4000 mm/s. The “hot” laser cutting parameters amounted to an average laser output power of 60%, a repetition rate of 300 kHz, a scanner velocity of 3000 mm/s with 2000 runs per sample, with no pause for heat dissipation. This “hot” laser cutting process resulted in strong temper colors across the entire 1.4310 sample surface. The “medium” laser cutting process parameters amounted to 98%, 25 kHz, 1000 mm/s, 8 loops of 10 runs per sample, with 10 s of pause at the end of each loop for heat dissipation. Temper colors appeared only very close to the laser cut edges. The corresponding values for “mild” laser cutting parameters amounted to 98%, 25 kHz, 1000 mm/s, 8 loops of 10 runs per sample, with 30 s of pause at the end of each loop for heat dissipation. This resulted in barely visible temper colors at the laser cut edges. For annealing in an air atmosphere, a heat treatment oven (N 11/HR, Nabertherm GmbH, Lilienthal, Germany) was used. Stainless steel samples (1.4310, 1.4301) were annealed at 100 °C, 200 °C, 400 °C, or 600 °C, respectively, for one hour. Aluminum alloy EN AW-5005-H24 samples were annealed at 100 °C, 200 °C, or 400 °C, respectively, for one hour. The EN AW-6082-T6 samples were not subjected to further annealing heat treatments to avoid overaging.

Position-controlled micro-tensile tests were performed on a bench-top system (ZwickiLine Z2.5 TS, ZwickRoell GmbH & Co. KG, Ulm, Germany). It included a dedicated sample holder for miniaturized specimens equipped with alignment tools, adjustment screws, and precise mechanical guides. It further comprised a 2.5 kN force sensor (MEMS flex structure with piezoresistive strain gauges) and a laser extensometer (Figure 1b,c). In pre-tests, no statistically significant variation in measured material properties was observed within the strain rate range of 0.00025 s^−1^ to 0.0067 s^−1^ proposed in ISO 6892-1 [18].

For the microstructure analysis, the samples were mounted, polished and etched for 10 s. An Adler etching solution (DE-850108, Struers GmbH, Birmensdorf, Switzerland) containing 25 mL deionized water, 50 mL HCl, 15 g FeCl_3_ and 3 g ammonium tetrachlorocuprate(II) was mixed with nitric acid (70%), in a ratio of 2:1. Figure 2b shows a mounted, polished and etched sample of 1.4310 after the micro-tensile test. A microstructure analysis was performed using a scanning electron microscope (XL 30 ESEM, Philips, Eindhoven, The Netherlands). It was equipped with detectors for secondary electrons (SE), back scattered electrons (BSE) and an energy-dispersive X-ray spectroscopy (EDS, chemical microanalysis).

## 3. Results

### 3.1. Mechanical Strain–Stress Behavior of Miniaturized 1.4310 Samples

The first part of this investigation was focused on the high-strength austenitic stainless spring steel 1.4310. In the first set of experiments, the influence of sample microfabrication on the mechanical strain–stress behavior was studied. Two typical microfabrication methods for miniaturized 1.4310 components were selected. Laser cutting represented microfabrication techniques with heat introduction into the material. Abrasive microwaterjet cutting represented “cold” microfabrication technologies. In a second set of experiments, the effect of annealing on the strain–stress behavior of microwaterjet cut 1.4310 samples was investigated.

#### 3.1.1. Influence of Sample Microfabrication on the Strain–Stress Behavior of 1.4310

**Figure 3:**
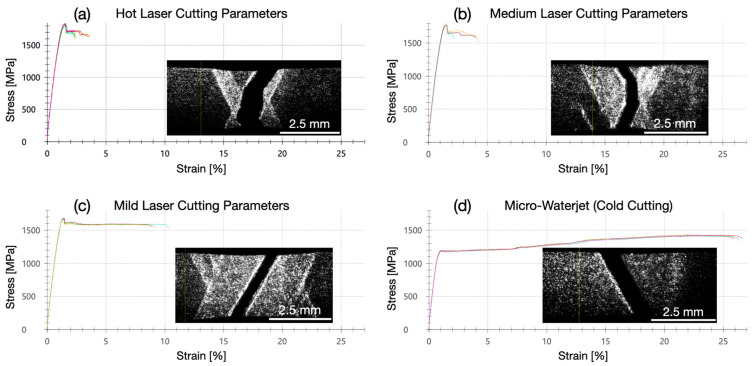
Micro-tensile tests of 100 μm thin 1.4310 steel. All laser cut samples failed due to the pronounced shear band zone formation at a low fracture strain, as shown in (**a**–**c**). The samples cut by the microwaterjet process exhibited local shear band zone formation only at the beginning of the plastic deformation (horizontal stress plateau), which evolved into a more homogeneous strain distribution over the entire measurement length and work hardening before the final fracture (**d**).

The micro-tensile tests shown in this chapter were performed with 1.4310 stainless spring steel samples at a strain rate of 0.0067 s^−1^. The laser cut samples failed due to the pronounced shear band zone formation and the early fracture within the shear band zones at a relatively low fracture strain. The occurrence of shear band zones could be observed in the laser extensometer images and in the horizontally oriented sections (stress plateaus) of the stress–strain curves (Figure 3a–c) at the beginning of the plastic deformation. In microwaterjet cut samples, plastic deformation also started by shear band zone formation (Figure 3d). It was, however, less locally confined, and rapidly evolved into a more homogeneous strain over the entire sample length including work hardening. It failed, at a relatively high fracture strain due to the shear fracture, under an angle of approximately 45°, with respect to the load axis.

**Figure 4:**
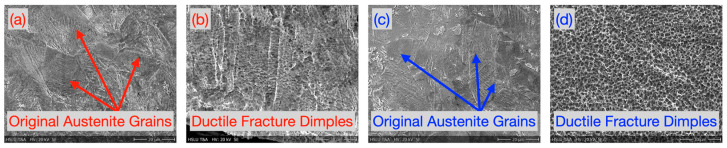
(**a**) Microstructure of the laser cut 1.4310 sample with polygonal, non-elongated grains, not deformed in a micro-tensile test. (**b**) Fracture zone of the laser cut 1.4310 sample with characteristic ductile fracture dimples after fracture in a micro-tensile test inside a shear band zone. (**c**) Microstructure of a microwaterjet cut 1.4310 sample with polygonal, non-elongated grains, not deformed in a micro-tensile test. (**d**) Fracture zone of a microwaterjet cut 1.4310 sample with characteristic ductile fracture dimples, after the fracture in a micro-tensile test.

**Figure 5:**
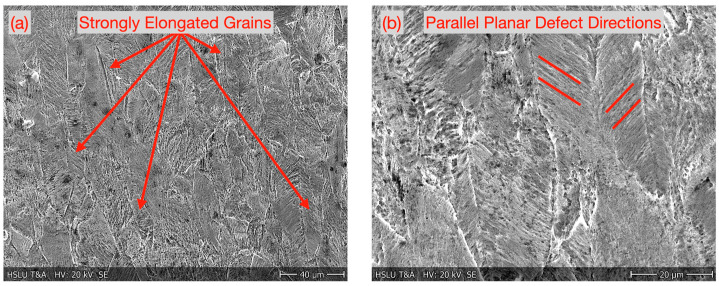
Microstructure of 1.4310 inside shear band zone. (**a**) Overview, grains strongly elongated by plastic deformation. (**b**) Detail with overlapping parallel planar defects in elongated grains.

**Figure 6:**
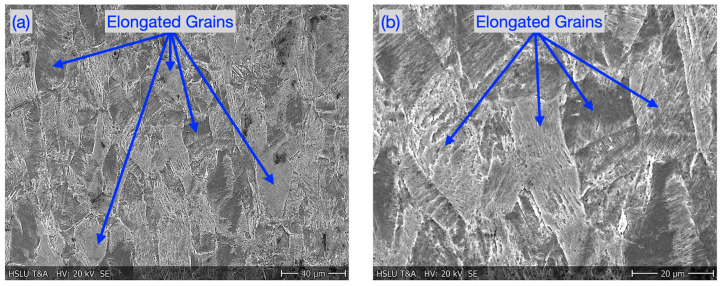
Microstructure of 1.4310 outside shear band zone; (**a**) overview and (**b**) in more detail.

The original austenitic grain microstructure of laser cut and microwaterjet cut 1.4310 samples (not deformed in micro-tensile tests) is shown in Figure 4a and Figure 4c, respectively. It was dominated by polygonal, non-elongated grains. This was in contrast to the microstructure with elongated grains in the deformation zone after micro-tensile tests (Figure 5 and Figure 6). Figure 4b and Figure 4c show the fracture zone of the laser cut and microwaterjet cut 1.4310 samples, respectively. Even the laser cut samples with low overall fracture strain but strong local plastic deformation in the shear band zone exhibited dimples. Dimples are characteristic for ductile fracture surfaces and evolve due to micro-void nucleation and coalescence.

Figure 5 shows the shear band zone microstructure of a 1.4310 micro-tensile test sample which was cut with hot laser parameters. The grain morphology in the shear band zone was significantly elongated by the plastic deformation. Numerous overlapping parallel planar defects appeared within the grains. According to [6], this indicates the formation of ε-martensite, stacking faults bundles, and twinning. Just outside the shear band zone, the grains were less elongated by plastic deformation (Figure 6).

For a quantitative characterization of the strain–stress behavior, the yield strength (R_p0.2_) at 0.2% plastic stain and the tensile strength (R_m_) at maximum stress and fracture strain were determined. The first maximum in the stress–strain curves was not treated as an upper yield strength (R_eH_) for the following reasons: Firstly, it was not a very sharp, discontinuous yield point at the end of a linear elastic slope. Such behavior would be typical for low-carbon unalloyed ferritic steels with body-centered cubic (bcc) structure. Secondly, the observed maximum was already well within the plastic region of the stress–strain curve, i.e., well beyond a plastic strain of 0.2%. It evolved after a continuous, steady transition from an elastic to plastic strain.

**Figure 7:**
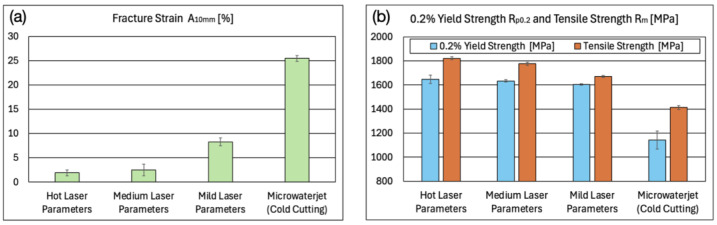
(**a**) Fracture strain A_10mm_, as well as (**b**) 0.2% yield strength R_p0.2_ (proof stress) and tensile strength R_m_ of 100 μm thin 1.4310 micro-tensile test samples microfabricated by pulsed fiber laser cutting and by cold microwaterjet cutting.

The fracture strain A_10mm_ amounted to 1.9% for the micro-tensile test samples cut with hot laser parameters and increased to 2.5% (medium laser parameters), 8.3% (mild laser parameters), and 25.4% (microwaterjet cutting). The 0.2% yield strength and tensile strength showed the reverse trend (Figure 7). The uniform strain A_u_ (i.e., the plastic strain at tensile strength) was not determined for the following reason: The occurrence of shear band zones, i.e., very locally confined strong plastic deformation, as well as the subsequent failure in the shear band zones impeded uniform plastic deformation over the entire measurement length in laser cut 1.4310 samples.

**Figure 8:**
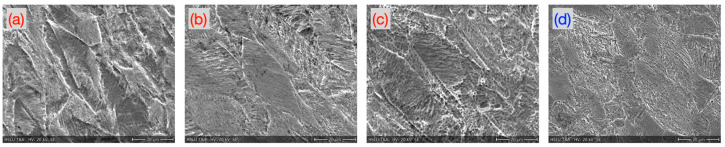
Comparison of the microstructure of the 1.4310 samples in the deformation zone (not in the shear band zones). Samples were cut with (**a**) hot, (**b**) medium, and (**c**) mild laser parameters, and with (**d**) microwaterjet. The laser cut samples (**a**–**c**) exhibited pronounced chromium carbide formation at the grain boundaries.

The laser cut samples exhibited a pronounced presence of chromium carbides at the grain boundaries, whereas the samples cut by the microwaterjet did not. These grain boundary chromium carbides appeared brighter in the SEM secondary electron images (Figure 8, deformation zone microstructure outside shear band zones). They resulted in elevated carbon signals in the qualitative local grain boundary EDS analysis.

#### 3.1.2. Influence Annealing Heat Treatments on the Strain–Stress Behavior of 1.4310

A defined quantity of samples produced by microwaterjet cutting was annealed in air at 100 °C, 200 °C, 400 °C, and 600 °C for one hour, respectively. Yellowish (at 400 °C) and bluish temper colors (at 600 °C) appeared. Temper colors on stainless steel surfaces consist of thicker oxide layers in which the chromium-to-iron ion ratio is considerably lower than in the 2–10 nm thin natural oxide. The reduced chromium-to-iron ion ratio depends on the heat treatment temperature and results in lower corrosion resistance [20,21].

**Figure 9:**
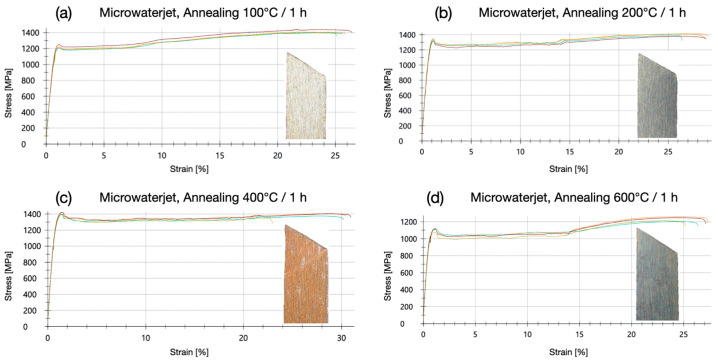
Strain–stress behavior and representative shear fracture images of 1.4310 micro-tensile test samples cut with microwaterjet and annealed at 100 °C (**a**), 200 °C (**b**), 400 °C (**c**) and 600 °C (**d**) for one hour, respectively.

The micro-tensile tests shown in this section were performed with annealed 1.4310 samples at a strain rate of 0.0067 s^−1^. Like in the microwaterjet cut samples without annealing (Figure 3d), plastic deformation started with slight shear band zone formation. It evolved subsequently into a more homogeneous strain distribution over the entire measurement length with work hardening. All samples failed by ductile shear fracture (Figure 9). In the stress–strain curves of the microwaterjet cut 1.4310 samples, without the heat treatment (Figure 3d) and annealed at 100 °C (Figure 9a), the horizontal shear band section amounted to below 10% of plastic strain. For samples annealed at 200 °C, 400 °C, and 600 °C, the horizontal shear band section extended to above 10% of plastic strain (Figure 9b–d).

**Figure 10:**
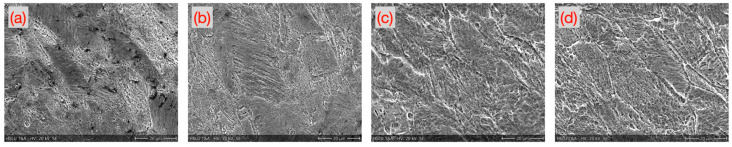
Deformation zone of 1.4310 samples cut by microwaterjet and annealed (**a**) at 600 °C, (**b**) at 400 °C, (**c**) at 200 °C, and (**d**) at 100 °C for 1 h, respectively.

Figure 10 shows representative images of the microstructure of the annealed 1.4310 samples after the micro-tensile tests. Chromium carbides appeared particularly often in the microstructure annealed at 600 °C. In general, the annealed microwaterjet cut samples contained lower amounts of chromium carbides at the grain boundaries than the pulsed fiber laser cut samples. In all annealed samples, distorted grains were observed with parallel lines inside, indicating overlapping planar defects. The samples annealed at 400 °C, which showed the longest shear band section in the stain-stress curves (Figure 9c), exhibited particularly strong signs of plastic deformation in some grains of its microstructure (Figure 10b).

**Figure 11:**
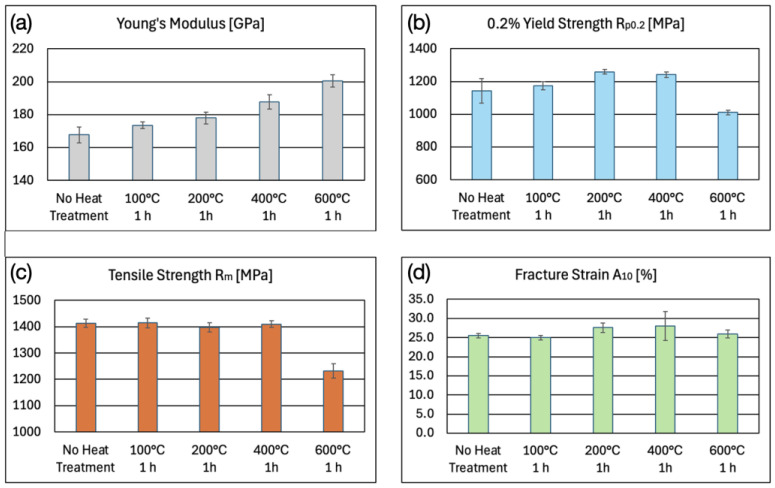
(**a**) Young’s modulus E, (**b**) 0.2% yield strength R_p0.2_ (proof stress), (**c**) tensile strength R_m_, and (**d**) fracture strain A_10mm_ of 100 μm thin 1.4310 micro-tensile test samples, produced by microwaterjet cutting, without and with annealing heat treatment.

Figure 11 depicts the mechanical properties of the microwaterjet cut 1.4310 samples with and without annealing. The 100 μm thin cold rolled samples contained a lot of lattice defects before annealing due to extensive work hardening. This resulted in a relatively low Young’s modulus of approximately 170 GPa. The annealing heat treatments increased the Young’s modulus, as shown in Figure 11a.

The determined values of R_p0.2_ at 0.2% plastic strain and of R_m_ at maximum stress are depicted in Figure 11b,c. Again, the first local maximum in the stress–strain curves of Figure 9 was not treated as an upper yield strength. It did not represent a very sharp, discontinuous yield point at the end of a linear elastic slope, like in the stress–strain curves of bcc low-carbon unalloyed steels. Instead, it was already well within the plastic region of the stress–strain curve, i.e., well beyond a plastic strain of 0.2%. The maximum occurred after a steady, continuous transition from elastic to plastic strain. It amounted to approximately 1200 MPa for non-annealed samples and for samples annealed at 100 °C. It changed to approximately 1300 MPa, 1400 MPa, and 1100 MPa for samples annealed at 200 °C, 400 °C, and 600 °C, respectively. The slight increase in R_p0.2_ (and of the first local stress maximum) until the annealing temperature of 400 °C could be related to the formation of first chromium carbides. Chromium carbides strengthen the grain boundaries and act as obstacles to dislocation glide inside the grains. The decrease in R_p0.2_ and R_m_ after annealing at 600 °C for 1 h was caused by the (partial) removal of the inherent work hardening effect from cold rolling. Stress relief annealing heat treatments of steels in industrial processes are typically performed at temperatures between 500 °C and 650 °C for one to several hours.

The fracture strain shown in Figure 11d did not change significantly as a result of the annealing heat treatments. What did change was the ratio of plastic strain caused by shear bands (i.e., the horizontal sections of the stress–strain curves in Figure 9) to the total amount of plastic strain. This ratio increased from approximately 23% (microwaterjet cut samples and samples annealed at 100 °C) to approximately 39% (annealing at 200 °C), up to around 58% (annealing at 400 °C), and then decreased again to approximately 44% (annealing at 600 °C). The decreased material strength after annealing at 600 °C did not result in an increased total fracture strain. The loss of cold work hardening may have been partially compensated for by chromium carbide formation which impedes plastic deformation.

### 3.2. Influence of Carbon Content and Stacking Fault Energy on the Strain–Stress Behavior

The second part of this investigation was focused on comparing the mechanical strain–stress behavior of miniaturized 1.4310 stainless spring steel samples to other fcc alloys. In a first set of experiments, the influence of carbon content on the deformation behavior of austenitic stainless steels was studied. For this purpose, micro-tensile tests were performed with microwaterjet cut and annealed samples of 1.4301 stainless steel. They exhibited a very similar chemical composition to 1.4310, except for a reduced amount of carbon. In the second set of experiments, the influence of stacking fault energy on the strain–stress behavior was investigated. Aluminum alloys with a significantly higher stacking fault energy than the austenitic stainless steels were chosen for this investigation.

#### 3.2.1. Influence of Carbon Content and Inherent Cold Work: 1.4310 vs. 1.4301

**Figure 12:**
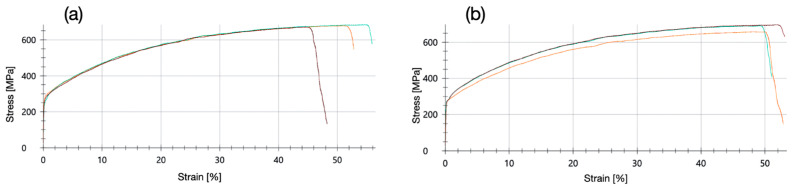
Micro-tensile tests curves of microwaterjet cut 1.4301 samples. No horizontal stress plateaus at beginning of plastic deformation. (**a**) No annealing. (**b**) After annealing at 600 °C for 1 h.

**Figure 13:**
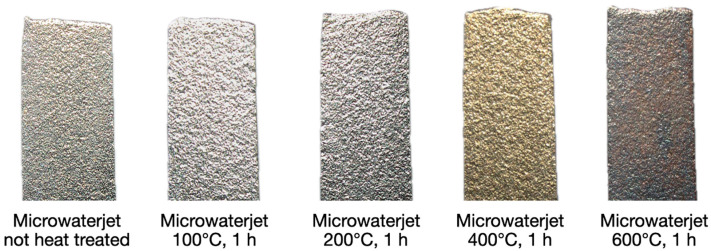
Microwaterjet cut 1.4301 samples (2.5 mm in width, 100 μm in thickness) of different annealing conditions, after failure in micro-tensile tests. All 1.4301 samples failed due to ductile fractures. Necking occurred mainly in the form of specimen thickness reduction at the fracture site.

The annealing heat treatments and micro-tensile tests shown in this chapter were performed with 1.4301 stainless steel samples containing 0.05% of carbon, in contrast to the 0.1% in 1.4310. There was no significant variation in other alloying elements [14,15]. A defined quantity of samples produced by microwaterjet cutting was subjected to annealing heat treatments at 100 °C, 200 °C, 400 °C, and 600 °C, for one hour, respectively. In the micro-tensile tests, the 100 μm thin and very soft 1.4301 material was difficult to clamp. The measurements became less accurate compared to the stronger 1.4310 material, particularly with respect to Young’s modulus E. A slower strain rate of 0.00025 s^−1^ resulted in more accurate measurement values of E. In contrast to 1.4310, no pronounced shear band zones were observed in laser extensometer images of the low-carbon 1.4301 samples, regardless of their annealing state. Stress–strain curves indicated no horizontally oriented sections at the beginning of plastic deformation (no stress plateaus). Work hardening was observed before reaching the maximum stress (Figure 12). All 1.4301 samples failed due to ductile fractures with necking (Figure 13).

**Figure 14:**
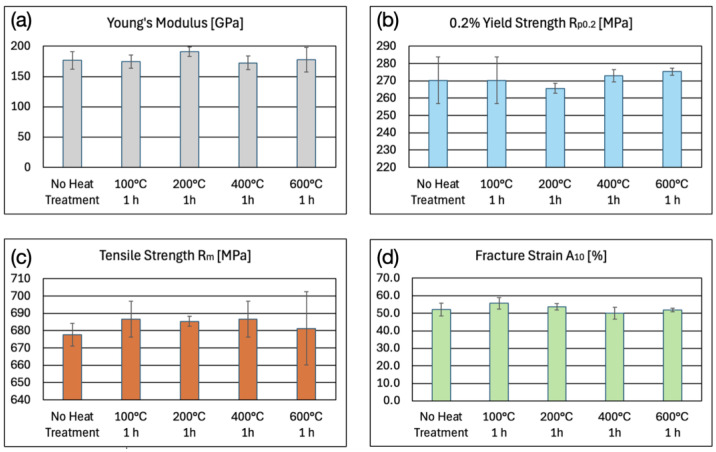
(**a**) Young’s modulus E, (**b**) 0.2% yield strength R_p0.2_ (proof stress), (**c**) tensile strength R_m_, and (**d**) fracture strain A_10mm_ of 100 μm thin 1.4301 micro-tensile test samples, produced by microwaterjet cutting, without and with annealing heat treatment.

Figure 14 depicts the mechanical properties of microwaterjet cut 1.4301 samples with and without annealing. No statistically significant influence of the heat treatments on Young’s modulus, tensile strength, and fracture strain was detectable. The slight increase in 0.2% yield strength after annealing at 400 °C and 600 °C may indicate some chromium carbide formation. Annealing did not cause an increase in Young’s modulus indicating relatively low amounts of inherent work hardening in the cold rolled samples.

Table 4 compares the mechanical properties of microwaterjet cut 1.4310 and 1.4301 samples, with rectangular cross-sections of 100 μm in thickness and 2.5 mm in width. According to [14], a measured tensile strength of (1413 ± 16) MPa for steel 1.4310 would correspond to an initial degree of deformation (inherent true strain) of approximately 40% from cold rolling. According to [15], the measured 0.2% yield strength and tensile strength depicted in Table 4 for steel 1.4301 would correspond to a rather low, below 10%, initial degree of deformation (inherent true strain). The relatively low initial degree of cold work in the tested 1.4301 samples is also reflected by the measured Young’s modulus (Figure 14a). It does not increase with the annealing temperature.

Another indication of the significant inherent work hardening in cold rolled 1.4310 was obtained by qualitative magnetic testing using rare earth permanent magnets. All 1.4310 micro-tensile test samples, regardless of their microfabrication and annealing state, exhibited ferromagnetic behavior. Ferromagnetic α′-martensite (forming martensite caused by plastic deformation) was already present in the cold rolled 100 μm thin 1.4310 raw material. On the other hand, all of the softer austenitic 1.4301 micro-tensile test samples, regardless of their annealing state, did not show ferromagnetic behavior and hence contained little or no forming martensite.

#### 3.2.2. Influence of Stacking Fault Energy: 1.4310 vs. Aluminum Alloys

The goal of these measurements was to evaluate the influence of stacking fault energy of fcc metals on the strain–stress behavior of miniaturized tensile test samples. In contrast to austenitic stainless steels, aluminum alloys possess a higher SFE, favoring full dislocation glide over partial dislocations and the related stacking fault formation or twinning. Aluminum alloys are more sensitive to heat exposure than steels. The micro-tensile test samples were fabricated by microwaterjet cutting. Two technically important aluminum alloys were chosen for this study. The first alloy was EN AW-5005-H24, a solid solution and work-hardened aluminum alloy. It was not oversaturated with magnesium (3% Mg) for maintained long-term corrosion resistance. A defined quantity of EN AW-5005-H24 samples was subjected to heat treatments at 100 °C, 200 °C, and 400 °C, for one hour, respectively. They did not develop temper colors. The second alloy was EN AW-6082-T6, a precipitation hardened aluminum alloy containing silicon and magnesium. It was provided by an automotive industry supplier. It was not annealed to avoid overaging. However, it was used to compare the micro-tensile rest results to the results of the standard size tensile tests with drawn proportionality rods of the same material.

**Figure 15:**
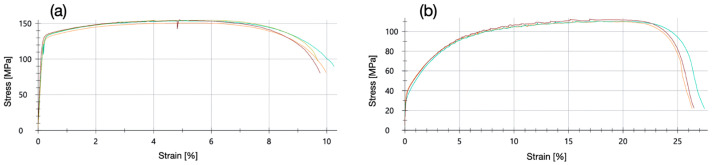
Micro-tensile test stress–strain curves of microwaterjet cut EN AW-5005-H24 samples. (**a**) No heat treatment; elevated R_p0.2_ due to significant work hardening effect after cold rolling. (**b**) After annealing at 400 °C for 1 h; low R_p0.2_ with subsequent work hardening.

**Figure 16:**
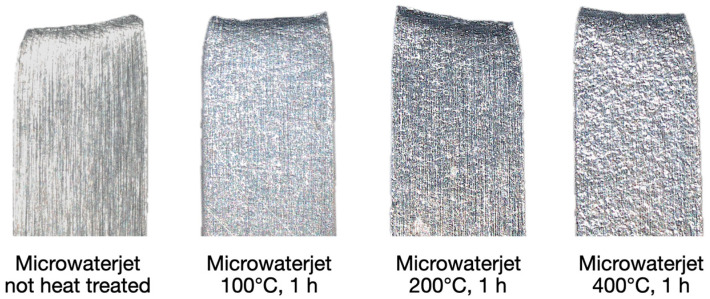
Microwaterjet cut EN AW-5005-H24 samples (3 mm in width, 500 μm in thickness) of different annealing conditions, after failure in micro-tensile tests. All samples failed by ductile fracture. Necking occurred in the form of pronounced thickness and width reduction at the fracture site.

The micro-tensile tests with EN AW-5005-H24 samples were performed at a strain rate of 0.00067 s^−1^. No pronounced shear band zones were observed in laser extensometer images. The samples annealed at 400 °C were rather soft and exhibited work hardening (Figure 15). All samples failed due to a ductile fracture with pronounced necking (Figure 16).

**Figure 17:**
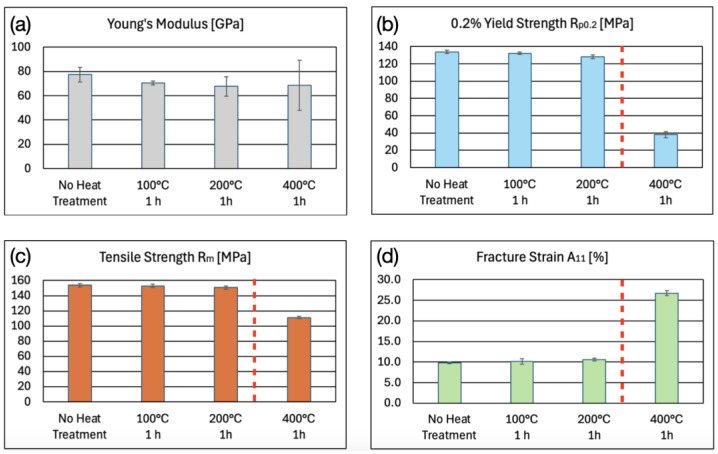
Mechanical properties of 500 μm thin microwaterjet cut EN AW-5005-H24 samples, without and with annealing (**a**–**d**). The decrease in 0.2% yield strength and tensile strength, and the increase in fracture strain after annealing at 400 °C were due to the removal of work hardening. The H24 partial annealing temperature of 260 °C is marked by the dashed red line in graphs (**b**–**d**).

The H24 treatment of alloy EN AW-5005-H24 comprised work hardening and partial annealing to half-hard at or slightly above a temperature of 260 °C [22]. After annealing at 400 °C, the work hardening effect disappeared because the heat treatment temperature was above the partial annealing temperature of the H24 treatment. A significant decrease both in 0.2% yield strength and in tensile strength, as well as a corresponding increase in fracture strain were observed after annealing at 400 °C (Figure 17).

**Figure 18:**
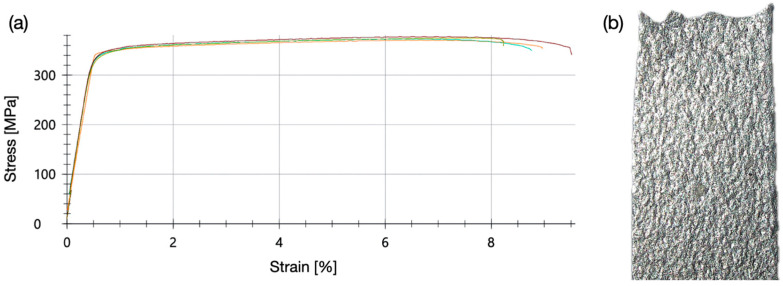
(**a**) Strain–stress behavior of the high-strength aluminum alloy EN AW-6082-T6, precipitation-hardened (artificial aging) and cut by microwaterjet. (**b**) The sample (3 mm in width, 1 mm in thickness) after a ductile failure in micro-tensile test.

In contrast to the solid solution and work-hardened alloy EN AW-5005-H24, aluminum wrought alloys of the type 6xxx are precipitation-hardened. The 1 mm thin EN AW-6082-T6 samples were cut by microwaterjet and tested at a strain rate of 0.0067 s^−1^. Alloy EN AW-6082-T6 was supplied in the artificially aged state for high material strength. After this heat treatment, large amounts of coherent precipitates and their elastic strain fields strongly interfere with complete dislocation glide and cross-slip. Despite these strong lattice defects, no shear band zone formation was observed in the laser extensometer images, nor the occurrence of a local stress maximum followed by a stress plateau at the beginning of plastic deformation in the stress–strain curves (Figure 18a). After slight work hardening, the samples failed by ductile fracture (Figure 18b). They did not exhibit the pronounced necking which was observed for the softer EN AW-5005-H24 samples.

The measured mechanical properties of EN AW-6082-T6 micro-tensile test samples and of round bar standard size tensile test samples are compared in Table 5. They comprise Young’ modulus E, 0.2% yield strength R_p0.2_, tensile strength R_m_. Fracture strain A_11mm_ was determined for the micro-tensile test samples (1 mm in thickness). Fracture strain A_5.65_ was determined for the standard size drawn round bar test samples (5 mm in diameter, proportionality factor of 5.65). The two different fracture strains which are related to the specific sample geometries permit only a qualitative comparison of ductility.

The thinner micro-tensile test samples exhibited an increased material strength, a decreased fracture strain, as well as a slightly lower Young’s modulus. These differences in mechanical properties were likely caused by the inherent plastic strain (work hardening) due to the cold rolling fabrication process of the thinner samples. The gain in strength comes at the cost of a lower ductility (fracture strain). This should be considered in the design of the miniaturized or lightweight components with thin cross-sections made of EN AW-6082-T6, e.g., for electromobility applications.

## 4. Discussion

The hypothesis that low stacking fault energy facilitates shear band formation in miniaturized fcc components was confirmed in this investigation. Shear band occurrence was observed in laser extensometer images and stress–strain curves of the micro-tensile test samples made of austenitic stainless steel 1.4310 with low SFE, but not in aluminum alloys with high SFE. However, low SFE alone was not a sufficient condition for the occurrence of shear bands. The lattice defects introduced by (micro-)fabrication, alloying, or heat treatments also affected the strain–stress behavior. Lattice defects typically comprise solid solution atoms of carbon and other alloying elements, cold work, grain boundaries or precipitations and dispersions of other metallurgical phases. They represent the obstacles to dislocation glide and cross-slip. The microstructure of 1.4310 exhibited more of these lattice defects in contrast to the other studied stainless steel 1.4301 with low SFE.

Microfabrication had a significant influence on the deformation behavior of 1.4310 stainless steel. The strain–stress behavior of microwaterjet cut 1.4310 samples was characterized by slight local shear band formation at the beginning of the plastic deformation. The initial shear band zones could be observed in the laser extensometer images and manifested themselves in horizontal sections of the stress–strain curves. They evolved subsequently into a more homogeneous plastic strain distribution over the entire measurement length and work hardening before the final fracture at a relatively high fracture strain (compared to laser cut 1.4310 samples). In a study of W. Mao et al. on ultra-fine grained austenitic stainless steel 1.4301, similar stress plateaus and related shear bands at low temperatures (170 K, 77 K) could be attributed to the localized transformation of austenite to martensite at the beginning of plastic deformation. Dislocation glide and work hardening, both in the austenitic and martensitic phases, set in later and resulted, subsequently, in a more homogeneous plastic deformation [23]. A similar strain–stress behavior with localized shear bands has been reported by Steineder et al. for cold rolled steels alloyed with 4–10% Mn and 0.1% C [24]. They contained approximately 30% of the retained austenite for transformation-induced plasticity (TRIP).

The strain–stress behavior of pulsed fiber laser cut 1.4310 samples, on the other hand, was characterized by pronounced shear band zone formation and early failure inside the narrow shear band zones at relatively low fracture strain. This effect could be attributed to the presence of heat-induced chromium carbides in the microstructure of laser cut 1.4310 micro-tensile test samples. Chromium carbides typically start to form in the martensitic phase of stainless steels at temperatures around 100–250 °C. This effect is well known in the context of heat treatments, e.g., in the Swiss knife industry [25]. They also form in larger amounts at the grain boundaries of austenitic stainless steels with sufficiently high carbon content at temperatures between 450 °C and 850 °C. This grain boundary sensitization has been studied in the context of intercrystalline corrosion [26,27,28,29].

Hot laser cutting parameters facilitated the chromium carbide formation in 1.4310 samples the most. Miniaturized samples exhibited a small total heat capacitance. The heat introduced by pulsed fiber laser melt cutting could not dissipate due to the absence of a larger thermal mass (no self-quenching). The heat-affected zone expanded over the entire miniaturized sample, resulting in chromium carbides particularly at the grain boundaries. The cold microwaterjet technique did not lead to chromium carbide formation. Ultra-short pulse pico- or femtosecond laser micromachining systems generating little HAZ would generally be a technological alternative to short pulse micro- or nanosecond fiber laser cutting of miniaturized components or thin sheets [30,31,32]. However, such high-power ultra-short pulse laser systems with fixed, trepanning or scanner optics are still too cost-intensive for many industrial applications. They were therefore not considered as a viable alternative for sample fabrication in the context of this application-oriented investigation.

Chromium carbides strengthened the grain boundaries of fiber laser cut 1.4310 samples and acted as obstacles to plastic deformation inside the grains. This increased the overall material strength. On the other hand, their presence also significantly decreased material ductility. They impeded the dislocation glide inside the grains and shear stress transfer across grain boundaries. Plastic deformation occurred mostly in the narrow initial shear band zones, i.e., in locally confined regions of material weakening. They had to accommodate large amounts of plastic strain and were particularly narrow in 1.4310 samples cut with hot laser parameters. Material failure at low overall fracture strain (i.e., embrittlement) occurred typically where the different shear band zones intersected.

The multitude of observed parallel planar defects in the shear band zone microstructure was consistent with the results of Talonen and Hänninen [6]. According to their study, the overlapping stacking faults of parallel {111} austenitic slip planes resulted in ε-martensite, stacking fault bundles, and twinning within the shear bands. Furthermore, they reported an (ferromagnetic) α′-martensite formation at shear band intersections. A more detailed analysis of the shear band zone microstructure and the planar defects using transmission electron microscopy may be the subject of future studies. They could include a selected area electron diffraction for crystallographic analysis, an electron backscatter diffraction, and a high-resolution chemical analysis of microstructural phases, as described in [33] in the context of ceramic-reinforced lightweight composite materials for automotive applications. Theoretical non-crystallographic shear band models, however, may also be suited to explain the certain aspects of the observed deformation behavior of 1.4310. They are based on certain stress conditions and explain the plastic deformation of nanocrystalline or amorphous alloys or of metals with very high lattice defect density in general [5,34,35].

Stress relief annealing heat treatments of microwaterjet cut 1.4310 samples containing 0.1% carbon also resulted in chromium carbide formation. However, all 1.4310 pulsed fiber laser cut samples exhibited stronger heat-induced chromium carbide formation at the grain boundaries (and inside the grains) than any of the annealed 1.4310 microwaterjet cut samples. None of the microwaterjet cut 1.4310 samples exhibited as pronounced narrow shear band zones as the samples microfabricated by short pulse fiber laser cutting. There was a strong correlation between the increased presence of chromium carbides in the microstructure of 1.4310, particularly at the grain boundaries, and pronounced shear band zone formation leading to early failure in the narrow shear band zones at low fracture strain in the micro-tensile tests.

Stress relief annealing increased the Young’s modulus of 1.4310 samples due to the healing of lattice defects created by cold rolling or by abrasive microwaterjet cutting. This annealing effect has also been reported for other metallic alloys with significant amounts of inherent plastic strain [36,37,38]. Thermally induced chromium carbides from laser cutting cannot be eliminated by stress relief annealing below the solution temperature.

Both aluminum alloys did not show shear band formation in the micro-tensile tests—neither the solid solution and work-hardened EN AW-5005-H24, nor the precipitation-hardened EN AW-6082-T6. Particularly EN AW-6082-T6 contained a large amount of lattice defects, e.g., coherent precipitates with strong elastic strain fields due to artificial aging (T6). They impeded the full dislocation glide and cross-slip. The observed stain–stress behavior without shear band formation could, therefore, be attributed to the high SFE of aluminum alloys. For EN AW-5005-H24, annealing heat treatments below the partial H24 annealing temperature of 260 °C may be applied to remove some of the lattice defects due to cold rolling and abrasive microwaterjet cutting. Higher annealing temperatures resulted in significant changes in the mechanical properties. For EN AW-6082-T6, annealing is not recommended to avoid overaging of the alloy.

The developed micro-tensile test setup, sample designs and mechanical testing procedures are suitable for measuring the strain–stress behavior of miniaturized samples. Shear band formation and work hardening can be identified in the stress–strain curves. Shear bands were observed in all 1.4310 micro-tensile test samples. They manifested themselves as a horizontal stress plateau at the beginning of plastic deformation in the stress–strain curves which was preceded by a moderate first stress maximum. Local shear band zone formation could also be observed in the laser extensometer images. Similar stress–strain curves with stress plateaus related to localized shear band formation at the onset of plastic deformation were reported in [23,24].

The observed strain–stress behavior of 1.4310, however, was different from the discontinuous upper yield strength maximum which is typically found in stress–strain curves of unalloyed ferritic low-carbon steels. They exhibit a very sharp local stress maximum at the end of the linear elastic slope. The subsequent yield point elongation in bcc low-carbon steels is attributed to shear bands (Lüders bands) caused by Cottrell atmospheres and dislocation pinning. The work hardening maximum in the plastic region of the stress–strain curve defines the ultimate tensile strength of these low-carbon ferritic steels. For fcc austenitic steels like 1.4310, with shear band formation due to low SFE and planar defects, however, the determination of R_p0.2_ seems to be more appropriate. The transition from elastic to plastic deformation of the 1.4310 samples occurred in a smoother way. The observed steady, continuous first local stress maximum was already well within the region of plastic deformation, i.e., well beyond a plastic strain of 0.2%. It was not represented by a sharp, discontinuous yield point maximum at the end of a linear elastic slope. Furthermore, R_m_ could then always be defined as the maximum stress in the stress–strain curve, even if samples failed in the shear band zones before the start of work hardening. Such a non-conventional strain–stress behavior was observed for laser cut 1.4310 samples in this investigation. It should be discussed in the future whether a dedicated standard needs to be developed for micro-tensile tests, with respect to sample geometries and test procedures. For Young’s modulus measurements of very soft materials, alternative complementary methods are recommended.

## 5. Conclusions

Microfabrication technologies do have a significant influence on the mechanical properties and the strain–stress behavior of miniaturized components. Chromium carbides in the microstructure of miniaturized laser cut 1.4310 samples caused pronounced shear band zone formation and early failure at a low fracture strain.

Shear band formation in fcc metals and alloys with thin cross-sections is further facilitated by the combination of low SFE and high lattice defect density. This was particularly the case for austenitic stainless spring steel 1.4310 with low SFE, elevated carbon content, and high amount of work hardening due to cold rolling. In contrast, neither of the investigated aluminum alloys with high SFE exhibited shear band formation regardless of work hardening or precipitation hardening.

The strain–stress behavior of miniaturized components with thin cross-sections made of fcc alloys remains an important research topic. Austenitic stainless steels and aluminum alloys offer good material strength, corrosion resistance, formability, and recyclability. Micro-tensile tests are expected to find interesting applications in material research and product development. One potential field of applications comprises miniaturized components for micromechanical systems, mechanical watch movements, small valves or electrical contact materials. Another application area emerges in the context of weight reduction in (electro-)mobility, transportation, aerospace, or cases for portable consumer electronics and point-of-care devices.

## Figures and Tables

**Table 1:** Chemical composition of 1.4310 and 1.4301 (min.-max. in wt%; Fe: balance).

	C	Si	Mn	Cr	Ni	Mo	P	S
1.4310	0.05 0.15	- 2	- 2	16 19	6 9.5	- 0.8	- 0.045	- 0.015
1.4301	- 0.07	- 1	- 2	17.5 19.5	8 10.5	- -	- 0.045	- 0.03

**Table 2:** Chemical composition of EN AW-5005 and EN AW-6082 (min.-max. in wt%; Al: balance).

	Si	Fe	Cu	Mn	Mg	Cr	Zn	Ti
5005	- 0.30	- 0.70	- 0.20	- 0.20	0.50 1.10	- 0.10	- 0.25	- 0.015
6082	0.70 1.30	- 0.50	- 0.10	0.40 1.00	0.60 1.20	- 0.25	- 0.20	- 0.10

**Table 3:** Dimensions (in mm) of the micro-tensile test samples; a_0_ represents the material thickness.

Alloy	a_0_	b_0_	L_0_	L_c_	B	H	L_t_	R
1.4310	0.1	2.5	10	10.65	5.0	6.0	40.0	20.0
1.4301	0.1	2.5	10	10.65	5.0	6.0	40.0	20.0
5005	0.5	3.0	11	19.84	8.0	9.0	60.0	20.0
6082	1.0	3.0	11	15.79	8.0	8.4	52.0	20.0

**Table 4:** Mechanical properties ^1^ of 100 μm thin 1.4310 and 1.4301 samples, cut by microwaterjet.

Material	E [GPa]	R_p0.2_ [MPa]	R_m_ [MPa]	A_10mm_ [%]
1.4310	168 ± 5	1140 ± 80	1413 ± 16	25.5 ± 0.6
1.4301	177 ± 14	270 ± 14	678 ± 7	52 ± 4

^1^ Young’s modulus E, 0.2% yield stress R_p0.2_, tensile strength R_m_, and fracture strain A_10mm_.

**Table 5:** Mechanical properties of EN AW-6082-T6 measured in micro- and standard- tensile tests.

Thickness t Diameter D	E [GPa]	R_p0.2_ [MPa]	R_m_ [MPa]	A_11mm_|A_5.65_ [%]
t = 1 mm	66 ± 2	343 ± 3	374 ± 3	8.4 ± 0.5 ^1^
D = 5 mm	68.3 ± 1.2	299 ± 3	325 ± 3	13.3 ± 0.9 ^2^

^1^ Micro-tensile test sample: Fracture strain A_11mm_. ^2^ Standard size round bars: Fracture strain A_5.65_.

## Data Availability

The original contributions presented in this study are included in the article. Further inquiries can be directed to the corresponding author.

## Abbreviations

The following abbreviations are used in this manuscript:

A_i_	Fracture strain (index “i” corresponds to a particular sample geometry)
bcc	Body-centered cubic
E	Young’s modulus
EDS	Energy-dispersive x-ray spectroscopy (chemical microanalysis)
fcc	Face-centered cubic
HAZ	Heat-affected zone
MEMS	Micro-electro-mechanical system
R_eH_	Upper yield strength
R_m_	Tensile strength
R_p0.2_	0.2% yield strength
SEM	Scanning electron microscope
SFE	Stacking fault energy

## References

[1] Schröder C., Volkova O., Wendler M. (2022). Influence of strain rate on the tensile properties of metastable austenitic stainless CrNi and CrMnNi spring steels. Mater. Sci. Eng. A.

[2] El-Danaf E. (2012). Mechanical properties, microstructure and micro-texture evolution for 1050AA deformed by equal channel angular pressing (ECAP) and post ECAP plane strain compression using two loading schemes. Mater. Des..

[3] Rohatgi A., Vecchio K., Gray G. (2001). The influence of stacking fault energy on the mechanical behavior of Cu and Cu-Al alloy: Deformation twinning, work hardening, and dynamic recovery. Metall. Mater. Trans. A.

[4] Zhao Y.H., Liao Y.Y., Zhu Y.T. (2005). Influence of stacking fault energy on nanostructure under high pressure torsion. Mater. Sci. Eng. A.

[5] Jia N., Roters F., Eisenlohr P., Kords C., Raabe D. (2012). Orientation dependence of shear banding in face-centered-cubic single crystals. Acta Mater..

[6] Talonen J., Hänninen H. (2007). Formation of shear bands and strain-induced martensite during plastic deformation of metastable austenitic stainless steels. Acta Mater..

[7] Kelly A., Knowles K.M. (2012). Crystallography and Crystal Defects.

[8] Hertzberg R.W., Vinci R.P., Hertzberg J.L. (2021). Deformation and Fracture Mechanics of Engineering Materials.

[9] Murr L.E. (1975). Interfacial Phenomena in Metals and Alloys.

[10] Huang K., Logé R.E. (2016). A review of dynamic recrystallization phenomena in metallic materials. Mater. Des..

[11] Lu S., Hu Q.-M., Johansson B., Vitos L. (2011). Stacking fault energies of Mn, Co and Nb alloyed austenitic stainless steels. Acta Mater..

[12] Lu J., Hultman L., Holmström E., Antonsson K.H., Grehk M., Li W., Vitos L., Golpayegani A. (2016). Stacking fault energies in austenitic stainless steels. Acta Mater..

[13] Qin B., Bhadeshia H.K.D.H. (2008). Plastic strain due to twinning in austenitic twip steels. Mater. Sci. Technol..

[14] Acidur 4310 (1.4310). https://www.swisssteel-group.com/content-media/documents/Data-Sheets/Stainless-Steel/1.4310_de.pdf.

[15] Acidur 4301 (1.4301). https://www.swisssteel-group.com/content-media/documents/Data-Sheets/Stainless-Steel/1.4301_de.pdf.

[16] EN-AW-5005 (3.3315). https://facts.kloeckner.de/werkstoffe/aluminium/en-aw-5005/.

[17] EN-AW-6082 (3.2315). https://facts.kloeckner.de/werkstoffe/aluminium/3-2315/.

[18] (2019). Metallic Materials—Tensile Testing—Part 1: Method of Test at Room Temperature.

[19] (2024). Standard Test Methods for Tension Testing of Metallic Materials.

[20] Kämmerer B. (2012). Chemical Composition in Stainless Steel Surfaces (Oberflächenzusammensetzung von Chromstählen). Ph.D. Thesis.

[21] Schütze M., Roche M., Bender R. (2015). Corrosion Resistance of Steels, Nickel Alloys, and Zinc in Aqueous Media.

[22] Engler O. (2014). Texture and anisotropy in the Al–Mg alloy AA 5005—Part I: Texture evolution during rolling and recrystallization. Mater. Sci. Eng. A.

[23] Mao W., Gao S., Gong W., Kawasaki T., Ito T., Harjo S., Tsuji N. (2024). Martensitic transformation-governed Lüders deformation enables large ductility and late-stage strain hardening in ultrafine-grained austenitic stainless steel at low temperatures. Acta Mater..

[24] Steineder K., Krizan D., Schneider R., Béal C., Sommitsch C. (2017). On the microstructural characteristics influencing the yielding behavior of ultra-ne grained medium-Mn steels. Acta Mater..

[25] Rechsteiner A. (2022). Personal Communication.

[26] Trillo E., Murr L. (1999). Effects of carbon content, deformation, and interfacial energetics on carbide precipitation and corrosion sensitization in 304 stainless steel. Acta Mater..

[27] Yae Kina A., Tavares S.S.M., Pardal J.M., Souza J.A. (2008). Microstructure and intergranular corrosion resistance evaluation of AISI 304 steel for high temperature service. Mater. Charact..

[28] Mayo E.W. (1997). Predicting IGSCC/IGA susceptibility of Ni–Cr–Fe alloys by modeling of grain boundary chromium depletion. Mater. Sci. Eng. A.

[29] Tavares S.S.M., Moura V., da Costa V.C., Ferreira M.L.R., Pardal J.M. (2009). Microstructural changes and corrosion resistance of AISI 310S steel exposed to 600–800 °C. Mater. Charact..

[30] Kaiser M., Kumkar M., Russ S., Auerswald J., Mielke M. Single pass laser cutting of high-precision components: Femtosecond versus picosecond pulses. Proceedings of the 34th International Congress on Applications of Lasers & Electro-Optics (ICALEO).

[31] Ackerl N., Fisch G., Auerswald J., Wegener K. (2020). Evolution of microstructures on stainless steel induced by ultra-short pulsed laser ablation. Springer Nat. Appl. Sci..

[32] Auerswald J., Ruckli A., Gschwilm T., Weber P., Diego-Vallejo D., Schlüter H. (2016). Taper Angle Correction in Cutting of Complex Micro-mechanical Contours with Ultra-Short Pulse Laser. J. Mech. Eng. Autom..

[33] Yang J., Heogh W., Ju H., Kang S., Jang T.S., Jung H.D., Jahazi M., Han S.C., Park S.J., Kim H.S. (2024). Functionally graded structure of a nitride-strengthened Mg_2_Si-based hybrid composite. J. Magnes. Alloys.

[34] Anand L., Su C. (2007). A constitutive theory for metallic glasses at high homologous temperatures. Acta Mater..

[35] Su C., Anand L. (2006). Plane strain indentation of a Zr-based metallic glass: Experiments and numerical simulation. Acta Mater..

[36] Kumar A., Li D.Y. (2022). Clarification of the Puzzled Effects of Cold Work on Wear of Metals from the Viewpoint of Wearing Energy Consumption. Tribol. Lett..

[37] Yoshida F., Uemori T., Fujiwara K. (2002). Elastic–plastic behavior of steel sheets under in-plane cyclic tension–compression at large strain. Int. J. Plast..

[38] Li Q., Hua G., Lu H., Yu B., Li D.Y. (2018). Understanding the Effect of Plastic Deformation on Elastic Modulus of Metals Based on a Percolation Model with Electron Work Function. J. Miner. Met. Mater. Soc..

